# A conserved mechanism of sirtuin signalling through steroid hormone receptors

**DOI:** 10.1042/BSR20193535

**Published:** 2019-12-10

**Authors:** Henry K. Bayele

**Affiliations:** Department of Structural and Molecular Biology, Division of Biosciences, University College London, Darwin Building, Gower Street, London WC1E 6BT, U.K.

**Keywords:** coactivator, gonadal steroid hormones, nuclear receptor coregulator, signal transduction, Sirtuins

## Abstract

SIRT1 and orthologous sirtuins regulate a universal mechanism of ageing and thus determine lifespan across taxa; however, the precise mechanism remains vexingly polemical. They also protect against many metabolic and ageing-related diseases by dynamically integrating several processes including autophagy, proteostasis, calorie restriction, circadian rhythmicity and metabolism. These sirtuins are therefore important drug targets particularly because they also transduce allosteric signals from sirtuin-activating compounds such as resveratrol into increased healthspan in evolutionarily diverse organisms. While many of these functions are apparently regulated by deacetylation, that mechanism may not be all-encompassing. Since gonadal signals have been shown to regulate ageing/lifespan in worms and flies, the present study hypothesized that these sirtuins may act as intermediary factors for steroid hormone signal transduction. Accordingly, SIRT1 and its orthologues, Sir2 and Sir-2.1, are shown to be veritable nuclear receptor coregulators that classically coactivate the oestrogen receptor in the absence of ligand; coactivation was further increased by 17β-oestradiol. Remarkably in response to the worm steroid hormone dafachronic acid, SIRT1 reciprocally coactivates DAF-12, the steroid receptor that regulates nematode lifespan. These results suggest that steroid hormones may co-opt and modulate a phyletically conserved mechanism of sirtuin signalling through steroid receptors. Hence, it is interesting to speculate that certain sirtuin functions including prolongevity and metabolic regulation may be mechanistically linked to this endocrine signalling pathway; this may also have implications for understanding the determinative role of gonadal steroids such as oestradiol in human ageing. At its simplest, this report shows evidence for a hitherto unknown deacetylation-independent mechanism of sirtuin signalling.

## Introduction

Biological ageing is a highly conserved process in all organisms and is regulated by endocrine signalling [1,2]. In humans, it is the single most important risk factor for ill-health and frailty because it is invariably accompanied by complex chronic comorbidities such as cancer and heart disease [3]. Although contentious, the sirtuins are presumed to regulate ageing by coordinating diverse processes that include autophagy, proteostasis, genome stability, metabolism, circadian cycling and calorie restriction. Of the seven human sirtuin isoforms, SIRT1 is of the most interest because it confers healthy ageing by protecting against or delaying the onset of pathologies including Type 2 diabetes, the metabolic syndrome and Alzheimer’s disease. When overexpressed or activated in mice fed sirtuin-activating compounds (STACs), Sirt1 also prevents obesity, improves energy balance, insulin-sensitivity and glucose tolerance, limits the damaging effects of fatty foods and delays ageing [4–7]. Its ability to translate the health benefits of STACs [8] further qualifies SIRT1 as an ideal polypill target to treat multiple diseases combinatorially [9]. However, this has been stymied by a limited understanding of its complex biology as a hub protein [10].

SIRT1 substrates include histones, multitudinous transcription factors and nuclear receptors (NRs) such as the androgen receptor (AR), oestrogen receptor α (ERα), liver X receptor α and LXRα ([11]; see references therein). Although there is no direct evidence that SIRT1 regulates global gene expression by histone modification, all reports suggest that it controls gene activity by deacetylating transcriptional regulators. It activates some but represses others, e.g. whereas LXRα is activated, the AR and ERα are repressed in prostate and breast cancer cells respectively. In the case of ERα, however, the evidence is mixed because its deacetylation by SIRT1 induces repression even though the transcriptional capacity of ERα depends on it being acetylated [12,13]. Paradoxically, PGC-1α deacetylation can lead either to its activation or repression depending on context. Similar discrepancies have been noted for PPARγ whose repression by SIRT1 occurs not by deacetylation *per se* but by corepressor recruitment [14]. Furthermore, because SIRT1 appears to elicit the substrate-specificity of an enzyme [15], the number of genes that it can coregulate by deacetylation is limited. This may be further complicated by observations that sustained activation of Sir2 induces heritable gene silencing (a surrogate marker for histone deacetylation) and shortens yeast lifespan [16], suggesting that longevity regulation may involve alternating cycles of gene silencing and derepression (acetylation). Furthermore, work with deacetylase-defective Sirt1 mutant mice showed that some of its functions do not require deacetylation [17]. These conflicting data indicate that deacetylation may not be all-encompassing as a mechanism of gene regulation by sirtuins and that other pathways may yet exist.

The contribution of SIRT1 and its orthologues to lifespan extension is still vexing; while some find no link [18], others suggest that their prolongevity effect may be dosage-dependent [19]. A mouse model with brain-specific overexpression of *Sirt1* provides the only evidence to date of the link between Sirt1 and mammalian lifespan [20]. In *Caenorhabditis elegans* and Drosophila, although gonadal steroid hormones are involved in ageing and lifespan regulation [21–24], the precise signal transduction pathway has not been fully elucidated. The steroid receptor DAF-12 [25] regulates worm metabolism, reproductive development and lifespan by binding to the bile acid-like steroid hormones dafachronic acids, DAs, [23,24,26] but it is not known how signals from DAs/DAF-12 are transduced. In human ageing, steroid hormone signalling can also be inferred from the link between declining oestradiol levels and reproductive senescence (menopause) typified by a predisposition to cardiovascular disease, cognitive impairment and osteoporosis all of which are also linked to SIRT1. Amelioration of these conditions with oestrogen [27] may be further evidence of its involvement in healthy human ageing.

Based on the preceding, it was hypothesized that SIRT1 and related sirtuins may act as intermediary factors for steroid hormone signal transduction in a non-cell-autonomous manner. In addition and just as important was to provide context for how such an endocrine function might better present SIRT1 as a target for pharmacological intervention. Of all the results herein, the most salient reveals that SIRT1 and its orthologues in yeast (Sir2) and worms (Sir-2.1) use classical nuclear receptor (NR)-coregulator interactions [28] to signal through human and worm steroid receptors, and that sirtuin signalling can be modulated by oestradiol (hereafter referred to as E2 or oestrogen) and dafachronic acids respectively. These results strongly hint at a conserved endocrine mechanism in which steroid hormones may co-opt sirtuins to regulate pathways including ageing and metabolism. The surprising discovery that SIRT1 is also comparable to the prototypical ER coactivators PGC-1α and SRC-3/NcoA3 [29,30] not only shows that it is a coactivator in its own right but also suggests that some of its diverse functions may depend on this oestrogen-dependent circuitry. This new insight may help to reimagine oestrogen as a potential regulator of SIRT1 biology in health and disease.

## Materials and methods

### Reagents

17β-Oestradiol (E2/oestrogen) and analytical grade reagents were obtained from Sigma-Aldrich (Dorset, U.K.); Δ^4^-dafachronic and (25S)-Δ^7^-dafachronic acids (DAs) were purchased from Cambridge Bioscience (Cambridge, U.K.) and Insight Biotechnology Ltd (Wembley, London, U.K.) respectively. Stock solutions of these ligands were prepared in sterile DMSO (Sigma-Aldrich) at 1000× concentrations (100 µM E2 or 1 mM DAs) and stored in aliquots at −20°C. Restriction and modifying enzymes were purchased from New England BioLabs (Hitchin, U.K.).

### Plasmid constructs

VP16-ER alpha (# 11351) and VP16-ER beta long isoform (#11352), p413TEF-Sir2 (# 51742), 3xERRE/ERE-luciferase (# 37852), SIRT1.1 (# 13735), Flag-SIRT1 (# 1791), Flag-SIRT1 H^363^Y (# 1792) and Gal4-PGC-1 alpha (# 8892) were purchased from Addgene (Cambridge, MA, U.S.A.). pET28-Sir-2.1 was kindly provided by Leonard Guarente (MIT, Cambridge, MA, U.S.A.) while VP16-DAF-12 and its reporter gene *lit*-1K-TK-luc [23] were kindly offered by David Mangelsdorf (UT Southwestern Medical Centre, Dallas, TX, U.S.A.). SRC-3/NcoA3 was a kind gift from Véronique Azuara (Imperial College London, U.K.).

SIRT1 (Wt) and SIRT1 H^363^Y cDNAs were subcloned into the *Bam*HI*-Xba*I sites and in-frame with the Gal4 DNA-binding domain (Gal4DBD) vectors pFA-CMV (Agilent Technologies, U.K.) or pM (Clontech, France) to generate pFABD-SIRT1 (wt) and pM-SIRT1 H^363^Y respectively. pM-Sir2 was constructed by transferring the insert from p413TEF-Sir2 into the *Eco*R1-*Sal*I sites of pM. pFABD-Sir2.1 was constructed by shuttling Sir-2.1 from pET28-Sir-2.1 with *Pml*I-*Hind*III into the *Sma*I-*Hind*III sites of pFA-CMV; this construct lacks the first two amino acids of Sir-2.1. pFABD-SIRT1ΔNRB, lacking the nuclear receptor box (NR-box), was generated by site-directed mutagenesis (changing it from LKDLL to AKDAA) using the QuikChange Site-Directed Mutagenesis kit (Agilent Technologies). To generate Gal4DBD-STACS-AD/CCD (amino acids 82-507), pFABD-SIRT1 was digested with *Hin*dIII to remove the smaller fragment and religated. Gal4DBD STACs-AD Wt and Gal4DBD-STACs-AD ΔNRB were constructed by digesting pFABD-SIRT1 and pFABD-SIRT1ΔNRB respectively with *Bam*HI and *Mun*1 and ligating the inserts into the *Bam*HI/*Eco*RI sites of pFA-CMV. The positive control vector Gal4DBD-VP16 AD was constructed by subcloning a DNA fragment encoding VP16 AD peptide (YGALDMADFEFEQMFTDALGIDEYG) into the *Sma*I site of pM vector; orientation was confirmed by sequencing.

For protein expression, wild-type (Wt) and mutant (Mt) SIRT1 STACs-AD were subcloned from pFABD-SIRT1 and pFABD-SIRT1ΔNRB respectively as *Bam*H1-*Mun*I fragments into the *Bam*HI-*Eco*R1 sites of pGex6P-1 (GE Life Sciences, U.K.) to generate pGex6P-STACs-AD (Wt) and pGex6P-STACs-ADΔNRB respectively. Both constructs contained residues 82–243 of SIRT1 and were identical except for the leucine residues in the NR-box that were mutated to alanines (see above). pGex6P-SIRT1 (Wt) and pGex6P-SIRT1ΔNRB were constructed by subcloning inserts from pFABD-SIRT1 and pFABD-SIRT1ΔNRB respectively into the *Bam*HI-*Sal*I sites of pGex6P-1. Sir-2.1 cDNA was transferred from pET28a-Sir-2.1 as an *Nhe*I-*Xho*I insert into the *Xba*I-*Xho*I sites of modified pGex6P-1 to create pGex6P-Sir-2.1. pGex5x-Sir2 was constructed by ligating the insert from p413TEF-Sir2 into the *Eco*R1-*Sal*I sites of pGex-5x1 (GE Lifesciences).

To clone the wild-type NR-box of SIRT1, oligonucleotide duplexes encoding the peptide (RTILKDLLPET) were synthesized as follows:

SIRT1 NR-box/S: aattcCGAACAATTCTTAAAGATTTATTGCCGGAAACAggtaccg SIRT1 NR-box/A: gatccggtaccTGTTTCCGGCAATAAATCTTTAAGAATTGTTCGg.

The mutant NR-box peptide (RTIAKDAAPET) was encoded by the following oligonucleotides:

SIRT1 NRMt-box/S: aattcCGAACAATTGCCAAAGATGCGGCCCCGGAAACAggtaccg SIRT1 NRMt-box/A: gatccggtaccTGTTTCCGGGGCCGCATCTTTGGCAATTGTTCGg

To test whether NR-boxes (underlined) of SIRT1 orthologues in other organisms were functional, oligonucleotides encoding only the wild-type boxes were subcloned as follows:

1. Sirt1 of *N. furzeri* NR-box peptide: RAILRDLLPET.
  *Nf*Sirt1NRB/S: aattcCGAGCCATTCTAAGGGATTTGCTTCCTGAGACTggtaccg;
  *Nf*Sirt1NRB/A: gatccggtaccAGTCTCAGGAAGCAAATCCCTTAGAATGGCTCGg).
2. Sir2 NR-box peptide: IYYLIKLLGFE.
  Sir2NRB/S: aattcATTTACTATCTTATCAAGTTGCTAGGCTTTGAAggtaccg;
  Sir2NRB/A: gatccggtaccTTCAAAGCCTAGCAACTTGATAAGATAGTAAATg;
3. Sir-2.1 NR-box peptide: FAILSDLLERA.
  Sir-2.1NRB/S: aattcTTCGCCATATTAAGCGATCTTCTAGAACGTGCTggtaccg;
  Sir-2.1NRB/A: gatccggtaccAGCACGTTCTAGAAGATCGCTTAATATGGCGAAg.
4. dSir2 NR-box peptide: WDYLAHLLNEP.
  dSir2NRB/S: aattcTGGGACTATTTGGCCCACCTGTTGAACGAGCCGggtaccg;
  dSir2NRB/A: gatccggtaccCGGCTCGTTCAACAGGTGGGCCAAATAGTCCCAg.

All oligonucleotides were synthesized and purified by Eurofins Genomics (Ebersberg, Germany). *Eco*R1/*Bam*H1 cloning sites and a unique *Kpn*1 site are in lower case. Oligonucleotides were phosphorylated with T4 polynucleotide kinase, annealed in equimolar ratios to form duplexes as previously described [31,32] and ligated into the *Eco*R1 and *Bam*H1 sites of pM vector. Recombinant plasmids were identified by linearization with *Kpn*1 and further verified by DNA sequencing (Eurofins Genomics).

### Cell culture

All cell culture media and supplements except charcoal-stripped foetal bovine serum, CsFBS (Sigma-Aldrich) were obtained from Life Technologies (Paisley, U.K.). Hep3B (ECACC 86062703) and HEK293 (ECACC 85120602) were obtained from the European Collection of Authenticated Cell Cultures (ECACC) through Sigma-Aldrich. MCF-7 cells were kindly provided by Matilda Katan and Ivan Gout (University College London, U.K.). All cell lines (passage numbers <20) were routinely cultured in DMEM (with 25 mM HEPES, GlutaMAX-1 and 4.5 g/l glucose), supplemented with 10% FBS and antibiotics and antimycotics. For transfections, cells were passaged in phenol red-free DMEM supplemented with 10% CsFBS without antibiotics and antimycotics, and seeded at a confluence of 80–90% in 24-multiwell uncoated plates (Hep3B and MCF-7) or on BioCoat poly D-lysine plates (HEK293); both sets of plates were obtained from Corning (Appleton Woods, U.K.).

### Transcriptional activation assays

For mammalian one-hybrid assays, HEK293 cells were cotransfected with 100 ng/well each of the Gal4 reporter vector, pFR-Luc (Agilent Technologies), and Gal4BDB-SIRT1 Wt, Gal4DBD-STACs-AD Wt, Gal4DBD-STACs-ADΔNRB and Gal4DBD-STACs-AD/CCD. Two-hybrid assays were performed by transfecting HEK293 cells with 100 ng each of Gal4BDB-SIRT1, Gal4DBD-Sir2, Gal4DBD-Sir-2.1, Gal4DBD-PGC-1α or Gal4DBD-NR-box plasmid constructs for SIRT1, Sir2, Sir-2.1, dSir2 and *Nf*Sirt1, together with the activation domain vectors VP16-ERα or VP16-ERβ and pFR-Luc; the Gal4DBD vector pM was used as negative control for both one- and two-hybrid assays.

To ascertain SIRT1 NR-box specificity, ER+ breast cancer cells (MCF-7) were cotransfected with the ER reporter gene 3xERRE/ERE-luciferase and 100 ng each of Gal4DBD, VP16-ERα and VP16-ERβ; increasing amounts (50, 100 and 200 ng) of either the wild-type or mutant Gal4DBD-NR-box plasmids were cotransfected where necessary. For coactivation assays, VP16-ERα or VP16-ERβ was cotransfected into Hep3B cells with the ER reporter gene, without or with increasing concentrations of SIRT1, Sir2, Sir-2.1, SRC-3/NcoA3 and PGC-1α expression vectors. DAF-12 was similarly cotransfected with its reporter gene *lit-*1K-TK-luc alone or with the same coactivators. In all cases, 50 ng/well pSVβgal (Promega, U.K.) was cotransfected as internal control. Plasmids were diluted to the required concentrations in 200 µl phenol red-free Opti-MEM I and X-tremegene HP transfection reagent (Roche, U.K.) was added to a ratio of 1:3 as recommended by the manufacturer. Cells were transfected in a total volume of ∼ 500 µl complete phenol red-free DMEM; each sample was transfected in duplicate. Approximately 12 h after transfection, 500 µl fresh DMEM containing DMSO or 200 nM 17-β oestradiol and 2 µM DAs were added to the cells to give final concentrations of 0.1%, 100 nM and 1 µM respectively, and incubated for a further 24 h. Luciferase and β-galactosidase (βgal) activities were determined as previously described [31,32] with the luciferase and Beta-Glo assay reagents (Promega) respectively, and measured in white 96-well plates (Nunc, Denmark) using a Tropix TR717 microplate luminometer (Applied Biosystems, U.K.); βgal expression levels were used to normalize luciferase expression.

### Protein expression and purification

pGex6P-SIRT1, pGex6P-SIRT1ΔNRB, pGex6P-STACs-AD, pGex6P-STACsΔNRB, pGex6P-Sir-2.1 and pGex5x-Sir2 expression vectors or pGex6P-1 were transformed into BL21-CodonPlus (DE3)-RIPL competent cells (Agilent Technologies), and selected on LB/ampicillin (100 µg/ml) agar plates. Overnight cultures were grown from single colonies from each of the transformations in LB broth supplemented with ampicillin and 34 µg/ml chloramphenicol. For expression, the cultures were diluted 1:20 in 2X YTG medium (1.6% tryptone/1% yeast extract/0.5% NaCl/2% glucose) with antibiotics and grown to an OD_600_ of ∼0.6; optical densities were measured in a WPA CO8000 Biowave Cell Density Meter (Biochrom Ltd, Cambridge, U.K.). GST was expressed (from pGex6P-1) to provide a control in pull-down assays (see below) by inducing with 0.1 mM IPTG (Generon, U.K.) for 3 h at 37°C. For the sirtuins, the cultures were cooled on ice to ∼16°C and protein expression was induced by adding IPTG as above; the cells were incubated overnight at the same temperature in a Multitron orbital shaker (Infors HT, Switzerland). After centrifugation for 20 min at 10,000 rpm in a Beckman Avanti J-26 XP medium speed centrifuge, bacterial pellets were resuspended in Bugbuster supplemented with benzonase (both from Novagen, U.K.) and cOmplete protease inhibitor cocktail (Roche). Cell lysates were centrifuged for 30 min at 20,000 rpm and as most of the protein was insoluble (data not shown), the resulting pellets were dissolved in STE buffer (50 mM Tris pH 7.5/300 mM NaCl/1 mM EDTA/10% glycerol) containing 10% Sarkosyl [33], and then diluted to a final Sarkosyl concentration of 1% in STE buffer containing 2% Triton X-100/4% CHAPS (Cambridge Biosciences, U.K.). Proteins were purified with Glutathione Sepharose 4 Fast Flow (GE Healthcare) by a batch method. Briefly, cell extracts were incubated with resin for 5 h at 4°C on a rotary platform. The mixture was then transferred to Poly-Prep chromatography columns (Bio-Rad, U.K.), and unbound protein was collected in the effluent. After the resin had settled, columns were washed with 10 volumes of 1× GST Bind/Wash buffer (Novagen); recombinant proteins and GST were eluted with 50 mM Tris-Cl (pH 8.1)/50 mM reduced glutathione (Promega). Proteins were resolved on 4-12% NuPAGE Bis-Tris gels (Thermo Fisher Scientific, U.K.), and detected with Coomassie Blue R-250 stain (National Diagnostics, U.K.). Protein fractions were pooled, filtered through PD-10 columns (GE Healthcare) to remove glutathione, and eluted with exchange buffer (20 mM HEPES, pH 7.5, 150 mM NaCl, 0.1 mM EDTA, 10% glycerol).

### GST pull-down assays

For pull-down assays, ERα and ERβ were subcloned into pCITE-4a(+) (Novagen) to enhance cell-free expression. *In vitro* transcription and translation of the receptors were performed with the TNT T7 Quick Coupled Transcription/Translation system (Promega) for 90 min in the presence of [^35^S]-methionine (Perkin Elmer, U.K.) as instructed by the manufacturer. DAF-12 was similarly expressed but from the T7 promoter in the CMX vector backbone of VP16-DAF-12 [23]. As baits, partially purified GST, GST-SIRT1, GST-SIRT1ΔNRB, GST-Sir2, GST-Sir-2.1 and GST-STACs-AD wild-type and its NR-box mutant GST-STACs-ADΔNRB were immobilized on glutathione sepharose beads for 2 h at 4°C. Unbound proteins were removed by centrifugation and the beads were washed 3× with 1× GST Bind/Wash buffer and resuspended in 20 mM HEPES, pH 7.5, 150 mM NaCl, 0.1 mM EDTA, 10% glycerol. For interaction assays, 20–50 µl aliquots of sepharose slurry with bound proteins were incubated in binding buffer [(20 mM HEPES, pH 7.5, 150 mM NaCl, 0.1 mM EDTA, 10% glycerol, 0.05% NP-40, and cOmplete protease inhibitor cocktail (Roche)] in a total volume of 300 µl containing 5 µl ^35^S-labelled ERα or ERβ; DMSO (control) or E2 (1 µM final concentration) was added as necessary. Binding reactions were nutated for 2 h at 4°C. The sepharose beads were then washed 3× with 1× GST Bind/Wash buffer and immobilized protein complexes were eluted by resuspending the beads in 1 volume of 2× NuPAGE LDS sample buffer containing antioxidant and heating for 10 min at 70°C. After a brief spin at 13,200 rpm, 10 µl aliquots of each sample were resolved on 4–12% NuPAGE Bis-Tris gels alongside 0.5 µl of *in vitro* translated receptor protein and PageRuler Plus prestained protein ladder (ThermoFisher Scientific). Gels were fixed in 10% acetic acid/50% methanol for 1 h at room temperature with slow agitation on an orbital shaker, and subsequently soaked in 1% glycerol/7% methanol/7% acetic acid for 30 min. They were treated with Amplify (GE Lifesciences) as instructed, dried under vacuum at 80°C on a gel drier (Bio-Rad) and exposed to X-ray film (GE Lifesciences) at −80°C.

### Statistical analyses

Reporter gene expression data were analyzed with Microsoft Excel 2010, and graphs were plotted using GraphPad Prism version 5.04 (GraphPad Software, Inc., San Diego). All transfection experiments were replicated at least 3×, data points were plotted as means of duplicates ± S.E.M. Statistical significance was assessed using paired Student’s *t*-test to compare transcriptional outputs from DMSO- versus hormone-treated cells. Only *P* values ≤ 0.05 were accepted as statistically significant.

## Results

### SIRT1 contains an autonomous, autoinhibitory N-terminal activation function

**Figure 1:**
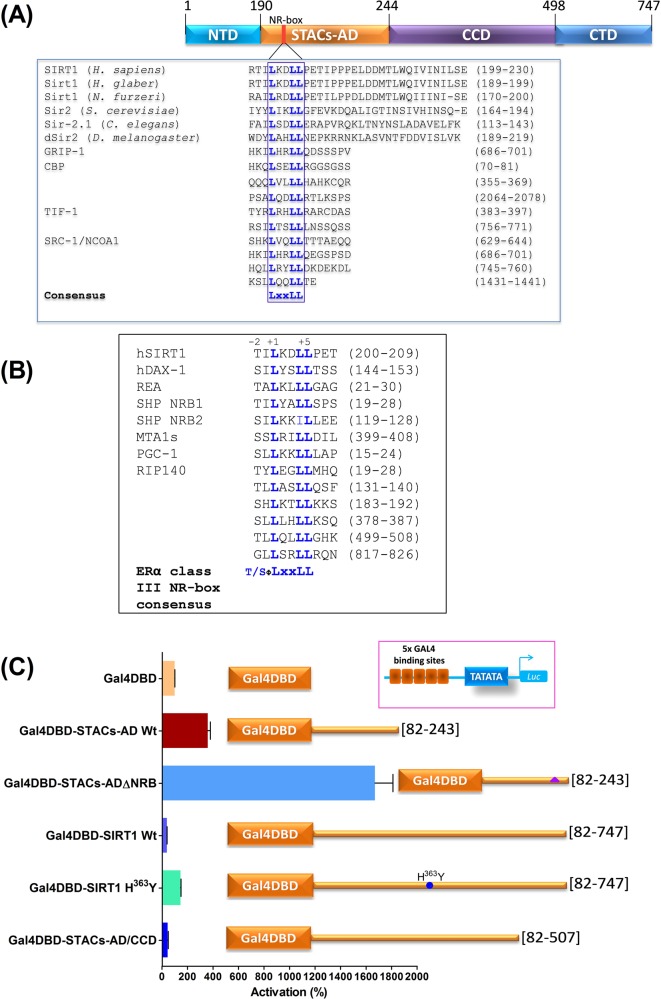
SIRT1 contains an autonomous, autoinhibitory N-terminal activation function (**A**) A simplified modular structure of human SIRT1 showing locations of the STACs-activation domain (STACs-AD) and the Core Catalytic (deacetylation) Domain (CCD). NTD and CTD are the N-terminal and C-terminal domains respectively. A classical nuclear receptor box (NR-box) is located within the STACs-AD. Position of the partially conserved STACs activation residue E230 is shown for context [15]. The alignment shows putative NR-boxes of SIRT1 (representing orthologues from different mammals) with orthologous sirtuins in the long-lived naked mole rat *H. glaber*, the short-lived killifish *N. furzeri, S. cerevisiae, D. melanogaster* and *C. elegans*. The NR-box core (LxxLL) of SIRT1 is homologous to those of prototypical coregulators GRIP-1 (Glucocorticoid Receptor-Interacting Protein-1), CBP (CREB-Binding Protein), TIF-1 (Transcriptional Intermediary Factor-1) and SRC-1/NCOA1 (Steroid Receptor Coactivator-1/ Nuclear Receptor Coactivator 1). (**B**) SIRT1 NR-box (see text) is a class III ERα-selective interface with a hydrophobic residue (Φ) at -1, and a hydrophilic residue (serine or threonine) at position -2 [41]. In (A) and (B), positions of the aligned sequences within the coregulators are bracketed. (**C**) One-hybrid assay in HEK293 cells to determine activation functions in SIRT1 and its derivatives tethered to the Gal4 DNA-binding domain (Gal4DBD) as shown. Each construct or Gal4DBD (pM backbone) was cotransfected with the minimal promoter vector pFR-Luc (containing 5x Gal4-binding sites, hereafter labelled 5x Gal4-luc) and β-galactosidase (β-gal) as reporter gene and internal control respectively. The transcriptional activities of the indicated constructs were calculated from the activity of Gal4DBD that was set at 100% as reference. 5x Gal4-luc expression levels were normalized with β-gal and plotted in duplicates as means ± S.E/M; they are representative of three separate experiments. Note that wild-type Gal4DBD-STACs-AD Wt and its mutant Gal4DBD-STACs-ADΔNRB are identical except for mutations in the NR-box (LKDLL→ AKDAA), shown with a triangle within this construct. The activity of Gal4DBD-SIRT1 Wt was compared with its deacetylase-defective mutant Gal4DBD-SIRT1 H^363^Y; mutation is shown with a blue dot. Bracketed numbers besides each construct indicate cloned regions (residues) of SIRT1.

SIRT1 has a unique intrinsically disordered N-terminal extension that appears to be critical for its enzymatic activity [34], regulation of gene transcription [35], and for binding STACs [15] as well as different effector proteins including histone H1 [36], AROS [37], necdin [38] and CLOCK [39], thus supporting its essential role as a molecular hub for diverse pathways [10]. A detailed examination of the STACs-AD (Figure 1A) revealed a single, classical nuclear receptor box, NR-box, [40] with the sequence LKDLL (where L is leucine, K is lysine and D is aspartic acid). This motif spans residues 202–206, upstream of E230 which mediates allosteric activation by STACs [15]. Unlike E230 however, the NR-box is highly conserved in all SIRT1 orthologues in *Saccharomyces cerevisiae, C. elegans* and Drosophila as well as in both the long-lived naked mole rat *Heterocephalus glaber* and in the short-lived fish *Nothobranchius furzeri*; they share the consensus sequence LxxLL (where x is any other amino acid) (Figure 1A). This NR-box is homologous to those of coregulators including RIP140/NRIP1, CBP/p300 and the steroid receptor coactivators (SRC-1/NCoA1, SRC-2/NCoA2/GRIP-1 and SRC-3/NCoA3/RAC3). Interestingly, it also belonged among the class III NR-boxes (Figure 1B) of the ER coregulators PGC-1α, REA, RIP140, MTA1s and the orphan NRs DAX-1 and SHP [30,41–43].

The possibility that SIRT1 might be a classical NR coregulator was initially tested for intrinsic activation function using the mammalian one-hybrid assay. Residues 82–747 and the STACs-AD (residues 82–243) were tethered to the yeast Gal4 DNA-binding domain (Gal4DBD) to generate Gal4DBD-SIRT1 or Gal4DBD-STACs-AD, respectively. Cotransfection into HEK293 with a Gal4 reporter gene showed that compared with Gal4DBD, Gal4DBD-STACs-AD increased reporter activity ∼4-fold (Figure 1C) but it was over 3000-fold weaker than Gal4DBD-VP16 activation domain (data not shown). In contrast, Gal4DBD-SIRT1 repressed Gal4 reporter activity as described [36], suggesting possible *cis*-dominant repression of the STACs-AD. In support of this, a chimaeric construct (Gal4DBD-STACs-AD/CCD; residues 82–507) coexpressing the STACs-AD and the core catalytic domain (CCD) was nearly as repressive as SIRT1; this indicates that the CCD contains the gene silencing function (Figure 1C). In contrast, a plasmid expressing the deacetylase-defective mutant Gal4DBD-SIRT1 H^363^Y was devoid of gene silencing capability. Paradoxically, Gal4DBD-STACs-ADΔNRB, a STACs-AD mutant in which the conserved leucine residues of the NR-box were changed (LKDLL→AKDAA), markedly activated Gal4 reporter activity ∼17-fold (Figure 1C) suggesting that the STACs-AD may be autoinhibitory. Since this was a one-hybrid assay, this result indicates that disruption of the STACs-AD rather mutation to the NR-box *per se* may have incidentally released autoinhibition, resulting in the increased activity shown. Taken together, these data reveal the STACs-AD to be an autonomous activation domain that may also be intrinsically autoinhibited. They are consistent with SIRT1 as a self-regulating protein [34], and with the widespread occurrence of autoinhibition in many proteins including kinases, transcriptional regulators, proto-oncogenes and the E3 ubiquitin ligase Parkin [44–46].

### Sirtuins interact with steroid receptors through conserved NR interfaces

**Figure 2:**
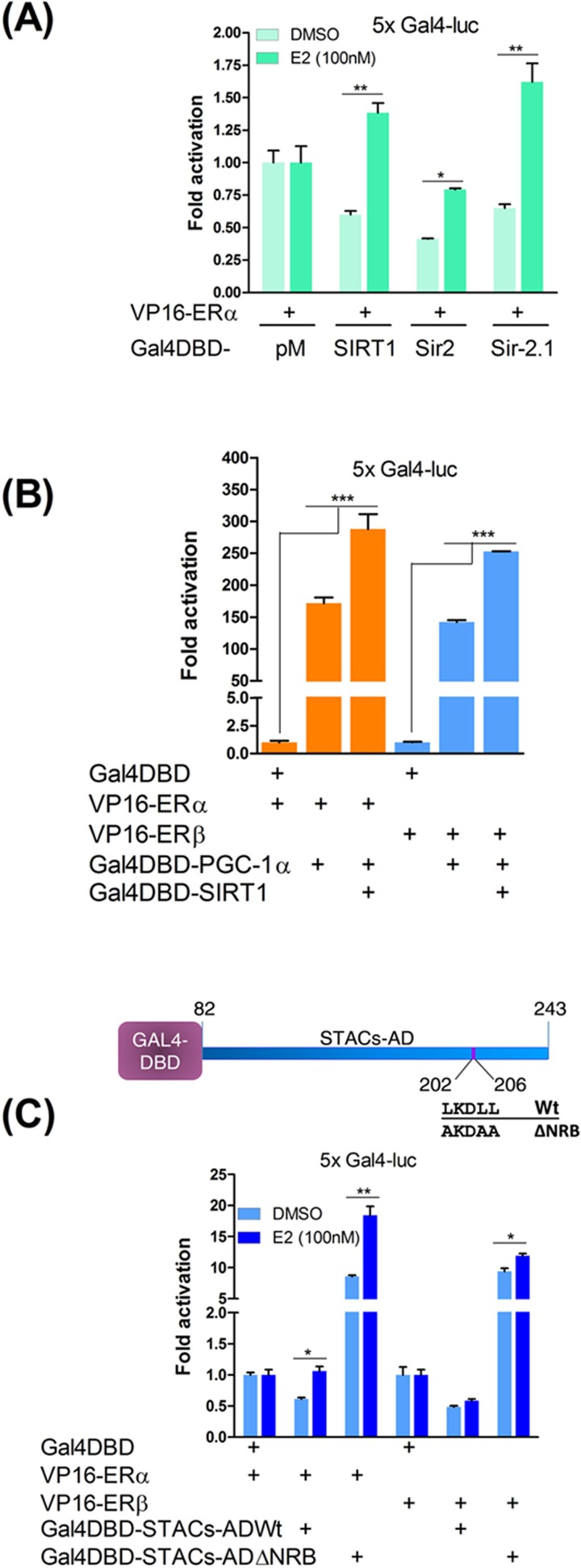
Mammalian two-hybrid (M2H) assays for ER interactions (**A**) M2H assays were performed in HEK293 cells to determine interactions between VP16-ERα and SIRT1, Sir2 and Sir-2.1 tethered to Gal4DBD. (**B**) SIRT1-PGC-1α transcriptional complexes strongly interact with ERα and ERβ and overide Gal4 promoter repression by SIRT1 alone. M2H assay was repeated with Gal4DBD-SIRT1 and Gal4DBD-PGC-1α, and ERα or ERβ fused to VP16 activation domain. (**C**) M2H assay for interaction between the STACs-AD and VP16-ERα or VP16-ERβ. Gal4DBD-STACs-AD Wt or its NR-box mutant (ΔNRB) were cotransfected with the receptors as indicated. Schematic shows locations of Wt and mutant NR-boxes within the STACs-AD. Statistical significance of differences in interaction (B) or gene expression in response to DMSO or E2 (A–C) are shown with *P* values: * *P* ≤ 0.05, ** *P* ≤ 0.01 and *** *P* ≤ 0.001; differences are not significant where *P* values are not shown. 5x Gal4-luc and β-gal activities were determined as described and ER interaction with Gal4DBD was set as reference point. Data were plotted in duplicates as means ± S.E.M and are representative of three independent experiments.

To determine sirtuin interaction with the ERs, Gal4DBD fusions of SIRT1, Sir2 and Sir-2.1 were tested in mammalian two-hybrid (M2H) assays. HEK293 cells were cotransfected with Gal4DBD or Gal4DBD-sirtuin fusion plasmids and VP16-ERα or VP16-ERβ. The data revealed that compared with Gal4DBD, interaction between the sirtuins repressed basal Gal4 reporter activity when bound to the ERs (Figure 2A). The reason for this is not known but is consistent with other findings that SIRT1 induces gene repression when targeted to Gal4-binding sites [36]. In contrast, PGC-1α strongly activated the reporter when bound to the receptors. Paradoxically, when SIRT1 and PGC-1α were co-expressed, they reported strong receptor interactivity (Figure 2B). While there could be a number of reasons for this, the most plausible explanation is that PGC-1α is a more powerful coactivator than SIRT1 (see below) which in addition to itself is capable of recruiting other (endogenous) coactivators, histone acetyltransferases and chromatin remodellers to promoters; it can also evict corepressors from these promoters [47]. Cumulatively, these functions probably over-compensated for Gal4 promoter repression by SIRT1. Wild-type STACs-AD also appeared to be constrained in its interaction with liganded-ERα or ERβ and was much less active than its NR-box mutant whose binding activated the reporter gene ∼17- and ∼20-fold, respectively (Figure 2C; cf Figure 1C). These results further indicate that the STACs-AD may be an autoinhibitory domain and that the increased activity of the mutant may be due to the release of autoinhibition rather than to ER binding *per se* since it bound poorly to the ERs relative to wild-type (see Figure 4C below and Discussion)*.*

**Figure 3:**
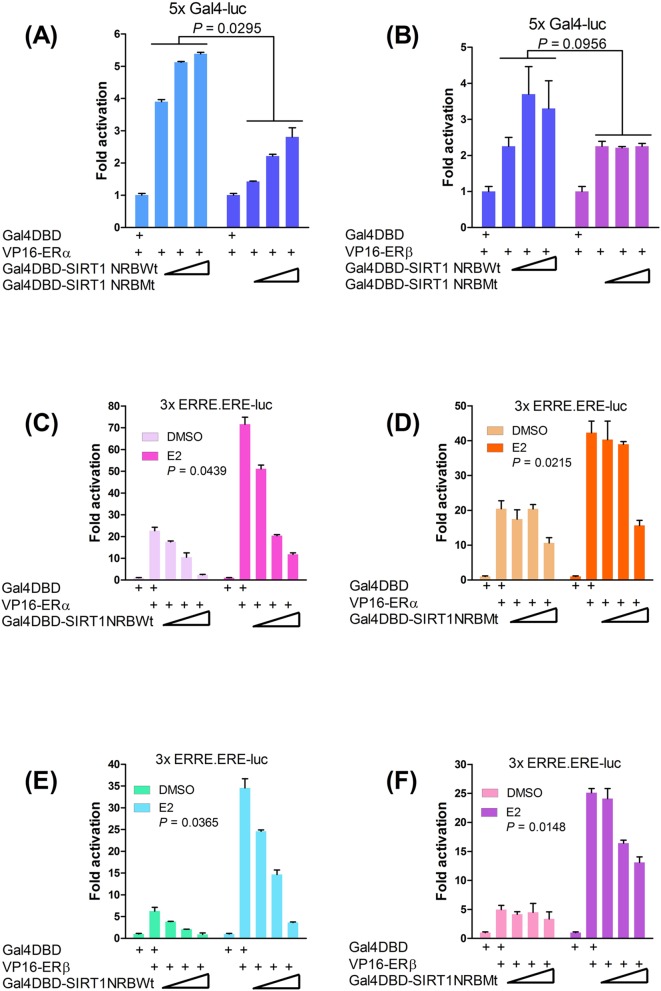
Determination of SIRT1 ER-binding specificity Increasing doses (50, 100 and 200 ng) of Gal4DBD fusion peptides of wild-type (NRBWt) and mutant (NRBMt) SIRT1 NR-boxes were tested in M2H assays in HEK293 cells for interaction with (**A**) VP16-ERα and (**B**) VP16-ERβ; the reporter gene was 5x Gal4-luc. Luciferase expression was normalized to β-gal activity. Graphs were plotted by setting the activity of Gal4DBD interaction with the ERs as reference. *P* values show the significance of differences in ER interaction, comparing NRBWt with NRBMt; only *P* values ≤ 0.05 are significant. (**C–F**) Modified M2H assay of SIRT1 NR-box peptide binding specificity by competitive inhibition with wild-type (C,E) and mutant (D,F) NR-box peptides. Interactions were determined through dose-dependent repression of ERα (C,D) and ERβ (E,F) signalling; antagonism was observed with wild-type (C,E) but not with mutant (D,F) NR-box peptides. For panels (C–F), MCF-7 cells were cotransfected with VP16-ERα or VP16-ERβ, the ER reporter gene instead of 5x Gal4-luc, and increasing amounts (50, 100 and 200 ng) of Gal4DBD SIRT1 NR-box peptides. Cells were treated with DMSO or 100 nM E2 and ER reporter gene expression was normalized to β-gal internal control. Fold activation was determined from the activity of Gal4DBD (negative control) set at a value of 1. *P* values show significant (*P* ≤ 0.05) differences between DMSO and E2-treated cells. Data are representative of three independent assays.

**Figure 4:**
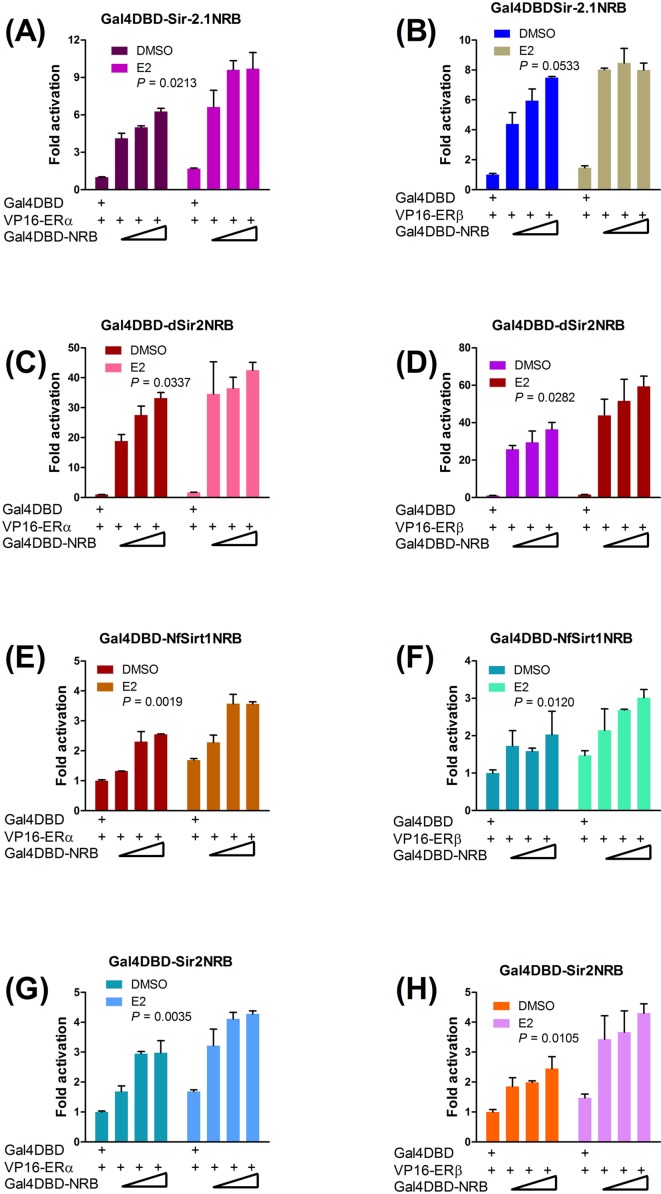
Conserved sirtuin NR-box peptides interact autonomously with the ERs Gal4DBD NR-box fusion peptides derived from *C. elegans* Sir-2.1 (**A,B**), Drosophila dSir2 (**C,D**), fish (*N. furzeri*) Sirt1, *Nf*Sirt1 (**E,F**), and *S. cerevisiae* Sir2 (**G,H**) were tested by the M2H assay for interaction with ERα (A, C, E, G) or ERβ (B, D, F, H). HEK293 cells were cotransfected with 5x Gal4-luc and increasing amounts of Gal4DBD NR-box (NRB) plasmids as indicated, or with Gal4DBD as negative control; cells were then treated with DMSO or 100 nM E2. In all cases, read-outs of ER interactions (fold activation) with the Gal4DBD were set as the reference point after normalizing luciferase activity against β-gal internal control. All data were plotted in duplicates as means ± S.E.M; data represent at least three separate experiments. Differences in gene expression between DMSO- and E2-treated cells were statistically significant if *P* ≤ 0.05.

Since NR-boxes are autonomous coactivator domains [37,38], wild-type SIRT1 NR-box peptide [RTILKDLLPET] and its mutant [RTIAKDAAPET] were expressed as Gal4DBD fusion peptides, with three residues on either side of the leucine-rich core to increase specificity. In M2H assays, SIRT1 NR-box interacted with both ER subtypes; mutating the conserved leucines diminished but did not eliminate receptor binding (Figure 3A,B). These results suggest that although the NR-box is necessary, flanking residues (see Figure 1A,B) may also be involved in ER binding consistent with other observations [41,48–50]. ER-binding specificity was further assured by a modified M2H assay using an ER instead of the Gal4 reporter gene. This showed that the wild-type peptide but not its mutant dose-dependently repressed ER transcriptional activity in the ER+ (MCF-7) breast cancer cells, indicating the potential of this peptide as an ER antagonist (Figure 3C–F). In further M2H assays to support conservation of the NR-box in the sirtuins, Gal4DBD fusions of the NR-box peptides of Sir2, Sir-2.1, dSir2 (Drosophila) and Sirt1 of *N. furzeri* (*Nf*Sirt1) showed ligand-independent binding to both ER subtypes that was further increased by E2 (Figure 4A–H). These data suggest that although the NR-boxes are sufficient for ER binding, they are not functionally equivalent. Interestingly, dSir2 NR-box peptide showed the strongest binding (Figure 4C,D), predicting similar interactions with an ER orthologue in Drosophila.

**Figure 5:**
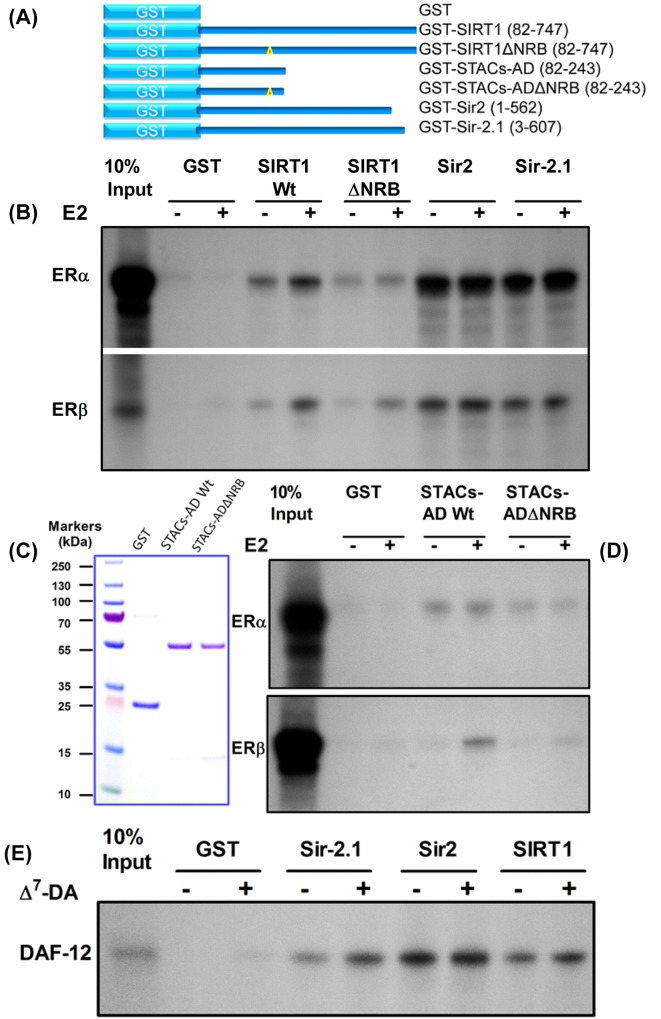
Reciprocal interactions between sirtuins and heterologous steroid receptors (**A**) Schematic of recombinant proteins used for interaction assays, depicting GST alone or fused to SIRT1 Wt, STACs-AD Wt, the NR-box mutants (SIRT1ΔNRB and STACs-ADΔNRB), Sir-2.1 and Sir2. Bracketed numerals show regions of the sirtuins expressed and yellow triangles indicate positions of deleted NR-boxes in SIRT1 and the STACs-AD. (**B**) Pull-down assays were performed with *in vitro* translated human ERα and ERβ by incubating with recombinant GST (control) or GST-SIRT1, GST-SIRT1ΔNRB, GST-Sir2 and GST-Sir-2.1 in the presence of 0.1% DMSO (-) to detect ligand-independent interaction or 1 µM oestradiol (E2). Bound proteins were detected by PAGE and autoradiography. (**C**) Coomassie Blue stain of purified recombinant GST, GST-STACs-AD Wt and GST-STACs-ADΔNRB; (**D**) Protein samples from (C) were incubated with ERα or ERβ; interaction was detected as in (B). (E) Sirtuin interaction with DAF-12. Binding reactions were performed with GST or GST fusions of Sir-2.1, Sir2 and SIRT1 (Wt only) incubated with *in vitro* expressed DAF-12 in the presence of DMSO (-) or 1 µM Δ^7^-dafachronic acid, DA (+). In all pull-down assays, the order of sample loading is as shown; all samples were processed in parallel on the same gel and binding data were derived from the same experiments (see Supplementary Figures S1–S5 for original images).

To confirm protein–protein interaction, pull-down assays were performed using recombinant GST or GST-tagged sirtuins (Figure 5A) and *in vitro* expressed ERα or ERβ. While GST did not bind to the receptors, GST-SIRT1 (Figure 5B) differentially bound both ERα and ERβ in the absence of ligand and E2 increased binding further. Interestingly, the NR-box was necessary for ligand-dependent ER binding as its mutagenesis markedly reduced SIRT1ΔNRB interaction in the presence of E2. However the mutations did not completely eliminate SIRT1ΔNRB binding, further suggesting that other residues at the NR interface may also bind to the receptors [41,48–50]. As expected, the STACs-AD was sufficient for interaction with both ER subtypes and E2 further increased binding (Figure 5C,D). Interestingly, these pull-down assays also showed strong ER binding to both Sir2 and Sir-2.1. Notably all three sirtuins bound to ERα more strongly than to ERβ. In further pull-down assays to test the universality of steroid receptor interaction, DAF-12 was chosen because of its role in nematode lifespan regulation [23,24]. The assays showed that all three sirtuins bound to DAF-12 ligand-independently and dafachronic acid (Δ^7^-DA) further increased binding (Figure 5E). As discussed below, the ability of Sir-2.1 to bind to DAF-12 in the absence of ligand was unexpected and may be physiologically relevant. Together, these data show that the sirtuins interact directly with steroid receptors through conserved interfaces.

### SIRT1 and its orthologues are veritable steroid receptor coactivators

**Figure 6:**
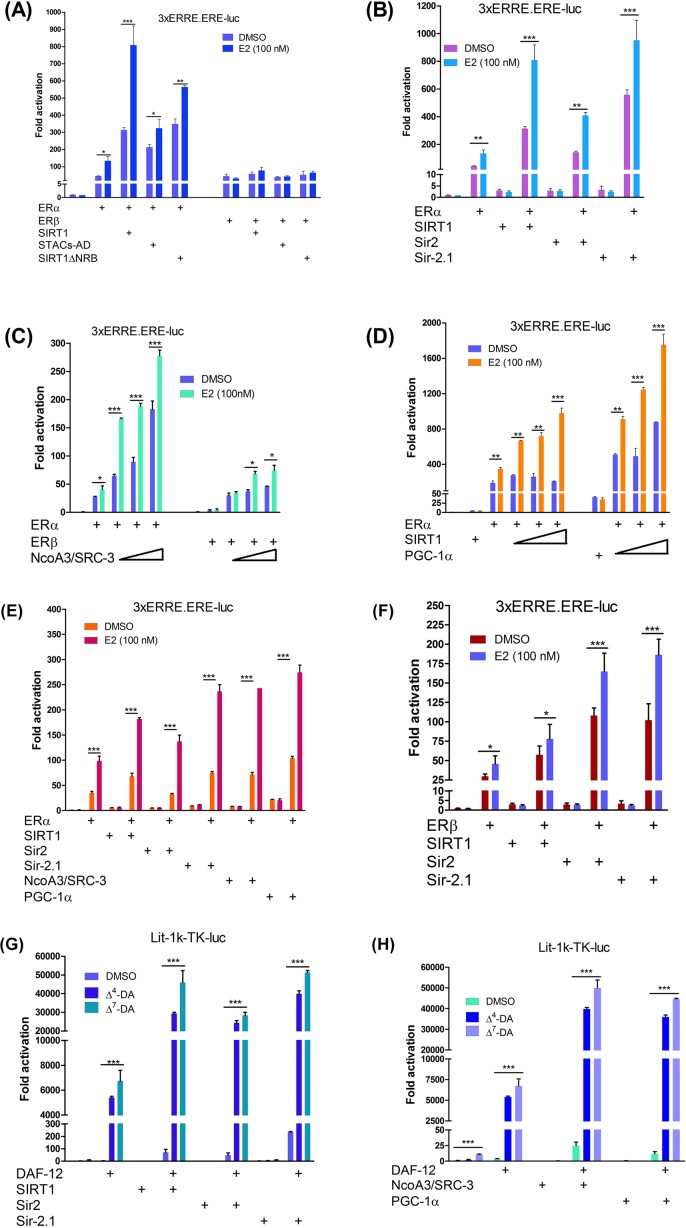
Conservation of sirtuin signalling through steroid hormone receptors (**A**) SIRT1 elicits ER subtype-selectivity in liver cells, coactivating ERα but not ERβ; the NR-box is required for ligand-dependent coactivation. Wild-type SIRT1 or its NR-box mutant SIRT1ΔNRB and the STACs-AD expression vectors were cotransfected into Hep3B cells with VP16-ERα or VP16-ERβ and the ER reporter gene 3xERRE.EREluc. (**B**) Sirtuins are veritable ER coactivators. Hep3B cells were cotransfected with 3xERRE.EREluc and SIRT1, Sir2 and Sir-2.1, with or without ERα. (**C**) ER subtype selectivity by SIRT1 is similar to NcoA3/SRC-3. (**D**) SIRT1 and PGC-1α are independent veritable ER coactivators. (**E**) Comparision of ERα coactivation by the sirtuins NcoA3/SRC-3 and PGC-1α. As in (**D**) note the strong intrinsic transcriptional activity of PGC-1α compared with the sirtuins and NcoA3/SRC-3. (**F**) Sir-2.1 and Sir2 but not SIRT1 show relaxed specificity towards ERβ. In (A–F) Hep3B cells were cotransfected with sirtuins and the ER reporter (3xERRE.EREluc) alone and together with ERα or ERβ. Ligand-independent coactivation was determined by incubating cells with DMSO while ligand-dependence was determined with 100 nM E2. (**G**), Reciprocal coactivation of DAF-12 by sirtuins. SIRT1, Sir2 and Sir-2.1 were coexpressed in Hep3B with the DAF-12 reporter gene (lit-1k-TK-luc) alone or together with DAF-12. Cells were treated with DMSO or 1 µM each of either Δ^4^- or Δ^7^-dafachronic acid (DA). (**H**), DAF-12 coactivation by sirtuins is comparable with NcoA3/SRC-3 and PGC-1α. For all samples (A–H), β-gal expression was used as internal control. All luciferase data were normalized to β-gal activity levels. Datasets were plotted in duplicates and shown as means ± S.E.M; each graph is representative of at least three independent experiments. The first pair of columns on each graph shows the activity of the reporter gene alone treated with DMSO or ligand. Where indicated, coregulators (sirtuins, NcoA3/SRC-3, and PGC-1α) were cotransfected with reporter genes alone to determine intrinsic transcriptional activity. The statistical significance of differences in gene expression between DMSO and ligand-treated cells are * *P* ≤ 0.05, ** *P* ≤ 0.01, and *** *P* ≤ 0.001; where none of these is shown, differences are not significant.

Conflicting reports suggest that NR regulation by SIRT1 may also involve deacetylation-independent mechanisms. To test this possibility as well as the functional relevance of the binding data, its ability to enhance ER transcriptional activity was measured. Cotransfection of an ER reporter gene and SIRT1 into Hep3B cells showed that alone, SIRT1 could not activate the reporter gene, indicating that it does not directly bind to DNA. However with the receptors it showed ER subtype-selectivity, strongly coactivated ERα and elicited ligand-independent coactivation that was further enhanced by E2; like SRC-3/NcoA3 [29], ERβ coactivation was comparatively weak (Figure 6A). These assays also showed reduced ligand-dependent coactivation by the NR-box mutant SIRT1ΔNRB whereas ligand-independent coactivation was unaffected (Figure 6A), indicating that this motif is necessary for oestrogen-dependent ER coactivation by SIRT1. Interestingly the STACs-AD was only ∼50% as effective as SIRT1, suggesting that maximal coactivation may require inter-domain interactions [34,35] with an ancillary activation region elsewhere within the protein.

Based on the binding data, it was hypothesized that SIRT1 and its orthologues shared a conserved steroid receptor signalling mechanism. To test if this was the case, ERα and ERβ were used as surrogate steroid receptors for *S. cerevisiae* and *C. elegans.* In cotransfections, Sir2 and Sir-2.1 also intriguingly increased unliganded and E2-dependent ERα transcriptional activation. As with SIRT1, both sirtuins differentially activated the ER reporter gene only in the presence of the receptors (Figure 6B), indicating that they are veritable ER coactivators. This was further confirmed by comparable ERα coactivation between the sirtuins and the prototypical ER coactivators, PGC-1α and SRC-3/NcoA3 (Figure 6C–E). Although PGC-1α deacetylation by SIRT1 is thought to underlie its metabolic functions [51], these results show that they may be independent ER coregulators. Of note, although PGC-1α was stronger than SIRT1, the latter elicited higher ligand-dependent ER coactivation because PGC-1α was constitutively more active than SIRT1 (Figure 6D,E). Hence in spite of their role in gene silencing, these sirtuins appear to be ER coactivators in their own right. Interestingly, whereas SIRT1 did not appreciably coactivate ERβ in this context, Sir2 and Sir-2.1 showed relaxed specificity towards this receptor subtype (Figure 6F). Together, these data show that despite the vast evolutionary distance between them, yeast and metazoan sirtuins use a conserved steroid receptor signalling mechanism, consistent with phylogenetic evidence that the ER is the ancestral steroid receptor [52].

The apparent conservation of this steroid receptor signalling mechanism predicted reciprocal coactivation of ER orthologues in other organisms by SIRT1, e.g. if Sir-2.1 could coactivate human ERs, would SIRT1 reciprocally coactivate worm steroid receptors? To test this possibility, Hep3B cells were cotransfected with DAF-12 and its reporter gene driven by the *lit-*1 kinase promoter [23], without or with each of the three sirtuins. Reporter assays showed that whereas they had no transcriptional activity of their own, they differentially coactivated DAF-12 in response to Δ^4^- and Δ^7^-DAs but not with DMSO (Figure 6G). These results reveal Sir-2.1 as a direct link between worm steroid hormones and DAF-12 signalling. They also show that whereas DAF-12 is almost entirely ligand-dependent, the ER elicits ligand-independent and ligand-dependent coactivation possibly because it has constitutively active and ligand-dependent domains. Crucially, both receptors were comparably coactivated by the sirtuins and SRC-3/NcoA3 or PGC-1α (Figure 6H), further confirming their status as steroid receptor coactivators. Together, these data suggest functional orthology between oestradiol and dafachronic acid on one hand, and the ER and DAF-12 on the other. They also suggest that steroid hormones may modulate a conserved mechanism of sirtuin signalling through steroid receptors.

## Discussion

SIRT1 and its orthologues have up till now been studied as epigenetic modifiers whose main mechanism of gene regulation is attributed to their deacetylation of histones and transcriptional regulators. However because acetylation/deacetylation is highly dynamic [53], incongruencies in sirtuin transcriptional outputs have arisen suggesting that deacetylation *per se* may not suffice to explain their functional pleiotropism. To resolve this conundrum, this report shows that these sirtuins are in fact veritable steroid receptor coactivators, providing a more classical mechanism of gene regulation than previously thought. Crucially, this new mechanism obviates the stringent specificity requirements of deacetylation substrates [15] or dependence on energy flux (NAD^+^/NADH ratios), and thus has the potential to massively expand the gene sets that these sirtuins may coregulate.

In a back-to-basics approach, the N-terminal STACs-AD of SIRT1 was tested for activation function based on the fact that that domain binds to different effector proteins involved in gene regulation. Consistent with this, it was found to contain a classical feature of NR coactivators, an NR-box [40] that is highly conserved in all SIRT1 orthologues from yeast to humans, in long-lived animals as well as in organisms with short lifespan. Reporter assays showed that when tethered to a heterologous DBD, the STACs-AD could activate gene expression, indicating that it is an autonomous activation domain. However, this assay also revealed a previously unseen regulatory mechanism in which the STACs-AD appears to be intrinsically autoinhibited, and in turn is repressed in *cis* by the CCD. This was based on data showing that although the STACs-AD activated Gal4 reporter gene expression on its own, this activity was repressed when it was coexpressed *in cis* but not in *trans* with the CCD (data not shown). Moreover, the STACs-AD became unexpectedly more active than the wild-type when it was disrupted by mutating the NR-box. This result suggests that the latter may be fortuitously positioned within an autoinhibitory domain, and that the increased activity in the STACs-AD mutant may be due to loss of autoinhibition. It is noteworthy that similar observations have been made in tightly regulated proteins including transcriptional regulators [44,45]. Interestingly, PGC-1α also has its NR-box within an inhibitory domain; like the STACs-AD, its NR-box mutant shows higher activity than wild-type PGC-1α [54]. These data are also consistent with reports of autoinhibition in other sirtuins [55,56]. Hence this mechanism may have teleological relevance e.g. in preventing spurious SIRT1 activation or for enabling selective binding to different effector proteins. It may also be physiologically important since loss of autoinhibition has been linked to several pathologies including the autoimmune syndrome, cancer, immunodeficiency and Parkinson’s disease [46,57–59]. Whether STACs-AD activation by STACs [15] is coupled to the release of autoinhibition would be interesting to examine.

The most important and potentially consequential outcome of the present study is that SIRT1 and its orthologues in yeast and worms differentially bound and coactivated human ERs, consistent with the ER as the primordial steroid hormone receptor [52]. All three sirtuins activated the ER reporter gene only in the presence of the receptors, indicating that they are ER coactivators *sensu stricto*. Interestingly SIRT1 also reciprocally coactivated DAF-12, suggesting that although DAF-12 is structurally more homologous to vertebrate sterol-activated NRs than to steroid receptors ([23]; see references therein), it may be functionally orthologous to the ER; this may be important because nematodes lost this receptor during evolution [52]. While ERα has previously been reported to be a deacetylation substrate, the new findings show that SIRT1 may also be a veritable ER coactivator. This is paradoxical given that coactivators such as SRC-3/NcoA3 have acetyltransferase activity whereas the sirtuins are deacetylases. It is presently unclear how SIRT1 may decouple coactivation from gene silencing but since these functions are performed by autonomous modules, ongoing work may resolve this dichotomy. It was also unexpected that the sirtuins were similar to prototypical ER coactivators. In particular, since SIRT1 was comparable to PGC-1α it may be able to coactivate ER-dependent genes without recourse to PGC-1α. On the other hand, their ability to form transcriptional complexes on the D-loop of mitochondrial DNA to which both ERα and ERβ bind [60,61] suggests that they may coactivate such genes synergistically (see Figure 2B).

These data may be informative on many levels but above all, they reveal the potential of steroid hormones to modulate key aspects of sirtuin biology including their roles in longevity and metabolism. While there is evidence that gonadal signals regulate ageing/lifespan, [21–24,26], whether those signals are transduced by the sirtuins remains to be tested. Nonetheless it is interesting to speculate that there may be cross-talk between the steroid signalling mechanism described herein and other pathways such as insulin/insulin-like growth factor-1 signalling and dietary restriction, DR, [62] to regulate sirtuin biology. For example, it has been shown that DR extends *C. elegans* lifespan by increasing Δ^7^-DA biosynthesis [63]. Although steroid signalling is found in yeast ([64]; see references therein), its link to yeast ageing is not known but on the basis of this dataset, it may be speculated that a Sir2-steroid receptor interaction system exists in this organism. In the case of DAF-12, its coactivation by Sir-2.1 may have direct relevance to their role in *C. elegans* lifespan and metabolic regulation. Paradoxically, these processes are also coregulated by DIN-1S, a DAF-12 corepressor that induces diapause [65]. Since Sir-2.1 could also ligand-independently bind to DAF-12 (see Figure 5E), it is interesting to speculate that it may still be able to coregulate DAF-12-dependent genes even when DA is limiting. Hence, further studies are needed to determine how this interplay may regulate nematode lifespan and/or metabolism.

Since extant steroid receptors are derived from a primordial ER [52], it could be speculated that sirtuin signalling may be coupled to functional ER orthologues in different organisms. In mammals, although the ERs are best known for their role in female development and reproduction, in both sexes they also regulate genes involved in energy balance, and lipid and glucose metabolism among others. Hence, they are linked to metabolic diseases and pathologies arising from declining oestrogen levels such as cardiovascular disease, cognitive impairment and osteoporosis [66,67]. Oestrogen is produced not only by the ovaries but also in non-reproductive tissues including bone, skin, liver, brain, pancreas, adipose tissue, skeletal muscle and the vascular endothelium. While these data remain to be tested in animal models, it is conceivable that locally synthesized oestrogen may modulate tissue-specific ER coactivation by SIRT1. Such studies may help to explain the overlapping disease phenotypes following ovariectomy [27,66], a pathologic naturally-occurring ERα deletion mutant that cannot bind oestrogen [68] and in ER and Sirt1 knockout mice; these conditions include insulin-resistance, the metabolic syndrome, Type 2 diabetes, obesity, infertility and loss of cognitive function [69–74]. Conversely Sirt1 overexpression prevents these conditions as well as ageing-related pathologies such as atherosclerosis, Alzheimer’s disease and osteoporosis [4–6,75]; it would be interesting to establish whether it does so by up-regulating ER signalling. Hence such information may improve our understanding of why the ER and SIRT1 are associated with the same metabolic and/or ageing-related diseases. Intriguingly, oestrogen and hypothalamic ERα have both been implicated in ageing [76,77] while long-term oestrogen therapy extended healthy lifespan in post-menopausal women [27]. Hence evidence that ER/oestrogen signalling regulates healthy human ageing is almost beyond question, but it remains to be established whether that requires SIRT1 co-optation.

In summary, this report has described a phyletically-conserved and previously unknown mechanism of sirtuin signalling involving classical nuclear receptor-coregulator interactions. This deacetylation-independent signalling pathway requires ligand-activated steroid receptors and thus directly links the sirtuins to endocrine signals (gonadal steroid hormones). It could provide an inkling into how sex hormones non-cell-autonomously regulate ageing, lifespan, metabolism and development and the role of the sirtuins in these processes [1,2,19–27,62,63]. Hence these findings augur well for further studies to determine whether oestrogen regulates healthy ageing by modulating sirtuin signalling through the ERs given their critical roles in health and/or protection from metabolic and ageing-related diseases [66,67]. Of immediate interest, the differential coactivation of ERα and ERβ by SIRT1 may present a molecular basis to test existing subtype-selective modulators against these diseases [78–80]. Hence, the present study may provide a mechanistic basis to underpin further study into how oestrogen may ameliorate ageing-related conditions or promote healthy ageing.

## Supplementary Material

Supplementary Figures S1-S5

## Abbreviations

CCD: core catalytic domain
CHAPS: 3-[(3-Cholamidopropyl)dimethylammonio]-1-propanesulfonate hydrate
DA: dafachronic acid
DBD: DNA-binding domain
DMEM: Dulbecco’s Modified Eagle Medium
E2: 17β-oestradiol
ER: oestrogen receptor
ERE/ERRE: ER/oestrogen-related receptor response element
FBS: foetal bovine serum
GST: glutathione *S*-transferase
IPTG: isopropyl β-D-1-thiogalactopyranoside
NcoA: NR coActivator
NR: nuclear receptor
PGC-1α: peroxisome proliferator-activated receptor gamma coactivator 1-alpha
Sir2: silent information regulator 2
SIRT1: silent information regulator 2 homologue 1
SRC: steroid receptor coactivator
STACs: sirtuin-activating compounds
STACs-AD: STACs-activation domain
