# Supplementary material for: A conserved mechanism of sirtuin signalling through steroid hormone receptors
Source: Biosci Rep. 2019 Dec 10;39(12):BSR20193535. doi: 10.1042/BSR20193535 (PMC6904774; doi:10.1042/BSR20193535)
Supplement: Supplementary Figures S1-S5 [file BSR-2019-3535_supp.pdf]

A conserved mechanism of sirtuin signalling through steroid hormone receptors

Henry K. Bayele

Department of Structural and Molecular Biology, Division of Biosciences, University College London, Darwin Building, Gower Street, London WC1E 6BT, United Kingdom

Correspondence:

Email: h.bayele@ucl.ac.uk

Tel: +44 207 6792454

Fax: +44 207 6797193

Supplementary Figures

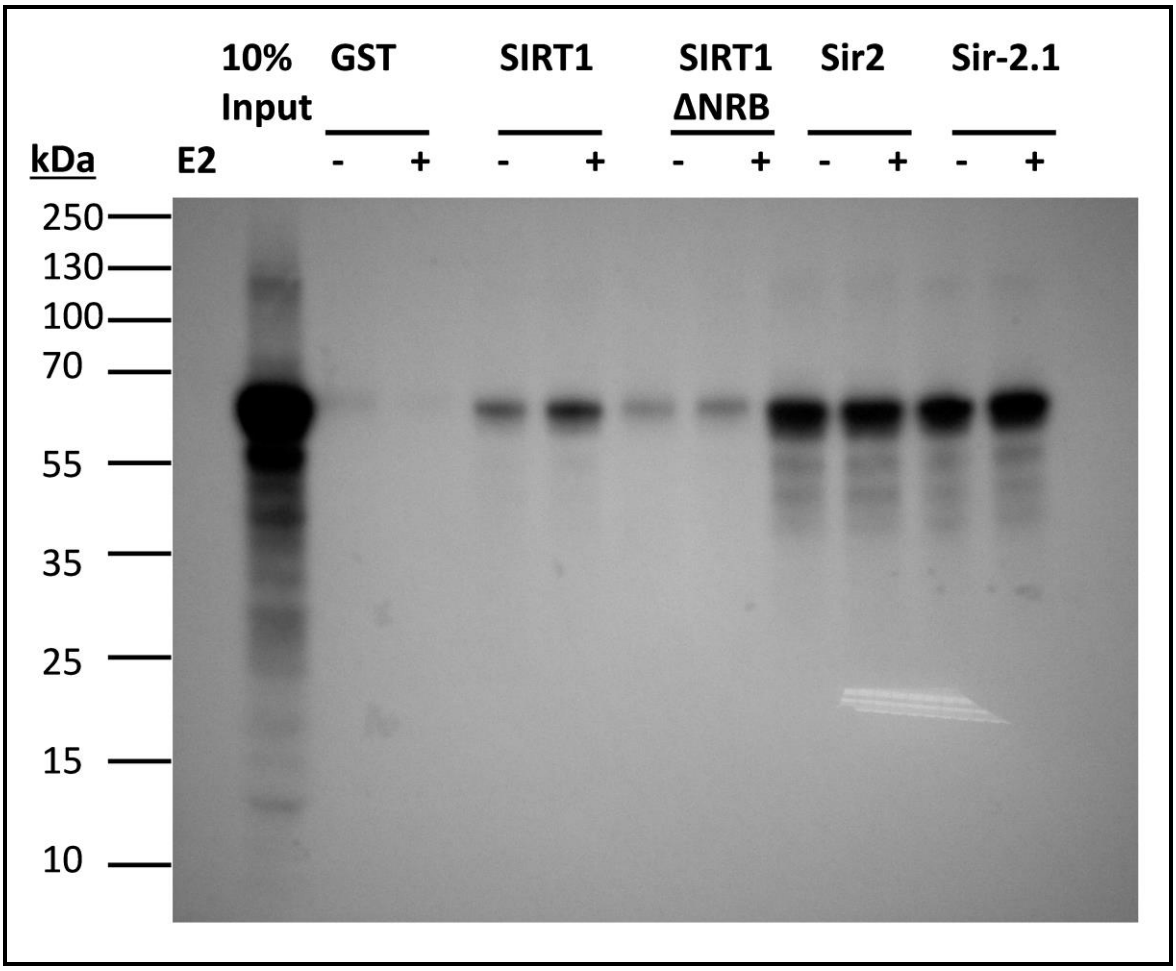

Figure S1. Assay of sirtuin interaction with ERα. Complete fluorograph of **Figure 5b** (upper panel). *In vitro* expressed ERα was bound to GST or GST-tagged SIRT1 (wt or NR-box mutant, ΔNRB), Sir-2.1 and Sir2 in the presence of DMSO (-) or 1 µM E2 to detect ligand-independent and ligand-dependent interaction respectively. Bound complexes were co-resolved with "Input" ERα and subjected to fluorographic detection; positions of molecular weight markers shown.

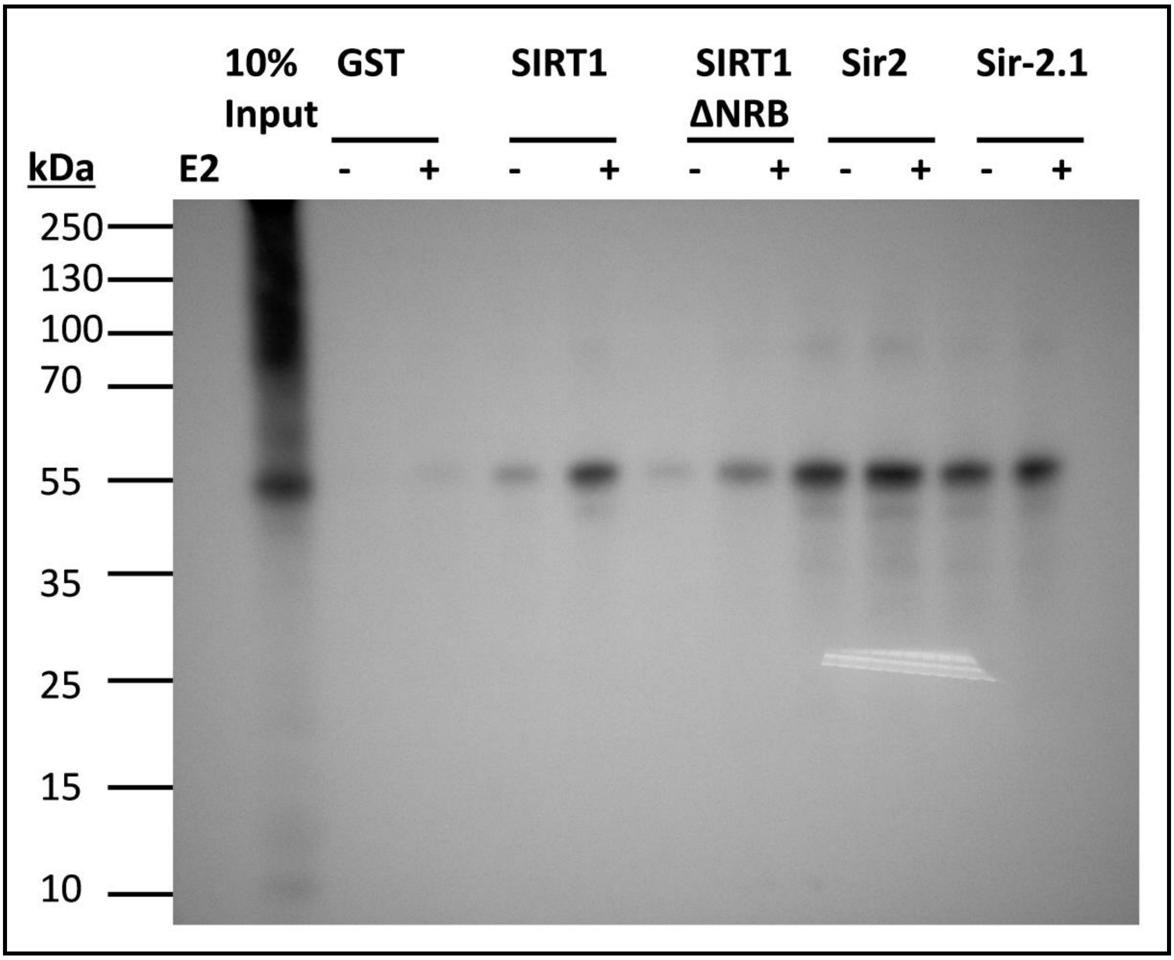

Figure S2. Interaction between sirtuins and ERβ. Full, uncropped fluorograph of **Figure 5b** (lower panel). *In vitro* expressed ERβ was bound to GST or GST-tagged SIRT1 (wt or NR-box mutant, ΔNRB), Sir-2.1 and Sir2 in the presence of DMSO (-) or 1 µM E2 to detect ligand-independent and ligand-dependent interaction respectively. Bound complexes were co-resolved with "Input" ERβ and subjected to fluorographic detection

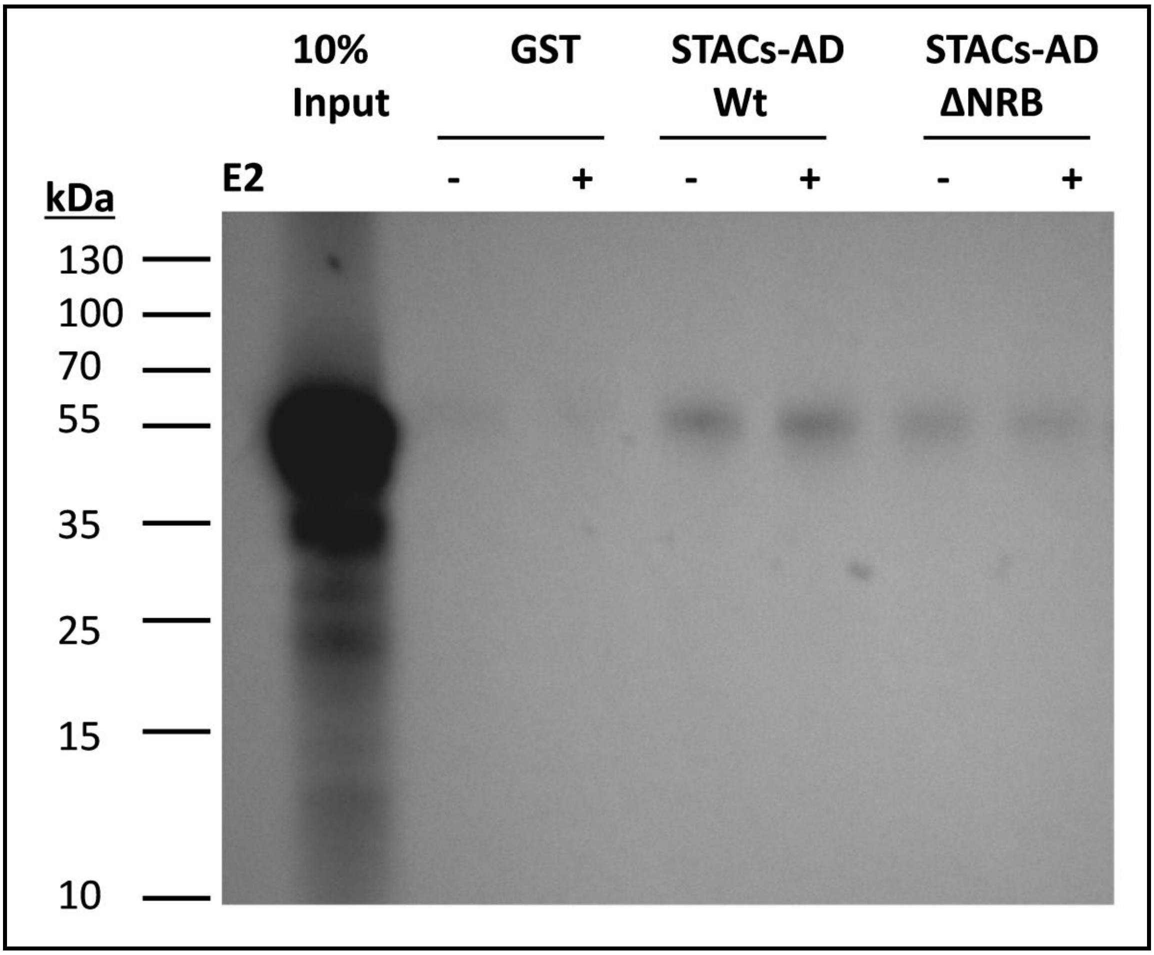

Figure S3. STACs-AD interaction with ERα. Uncropped fluorograph of **Figure 5d** (upper panel). *In vitro* expressed ERα was bound to GST or GST-tagged STACs-AD wt or its NR-box mutant (ΔNRB); DMSO (-) or 1 µM E2 were added to determine ligand-independent or ligand-dependent interaction respectively. Complexes were co-resolved with "Input" ERα.

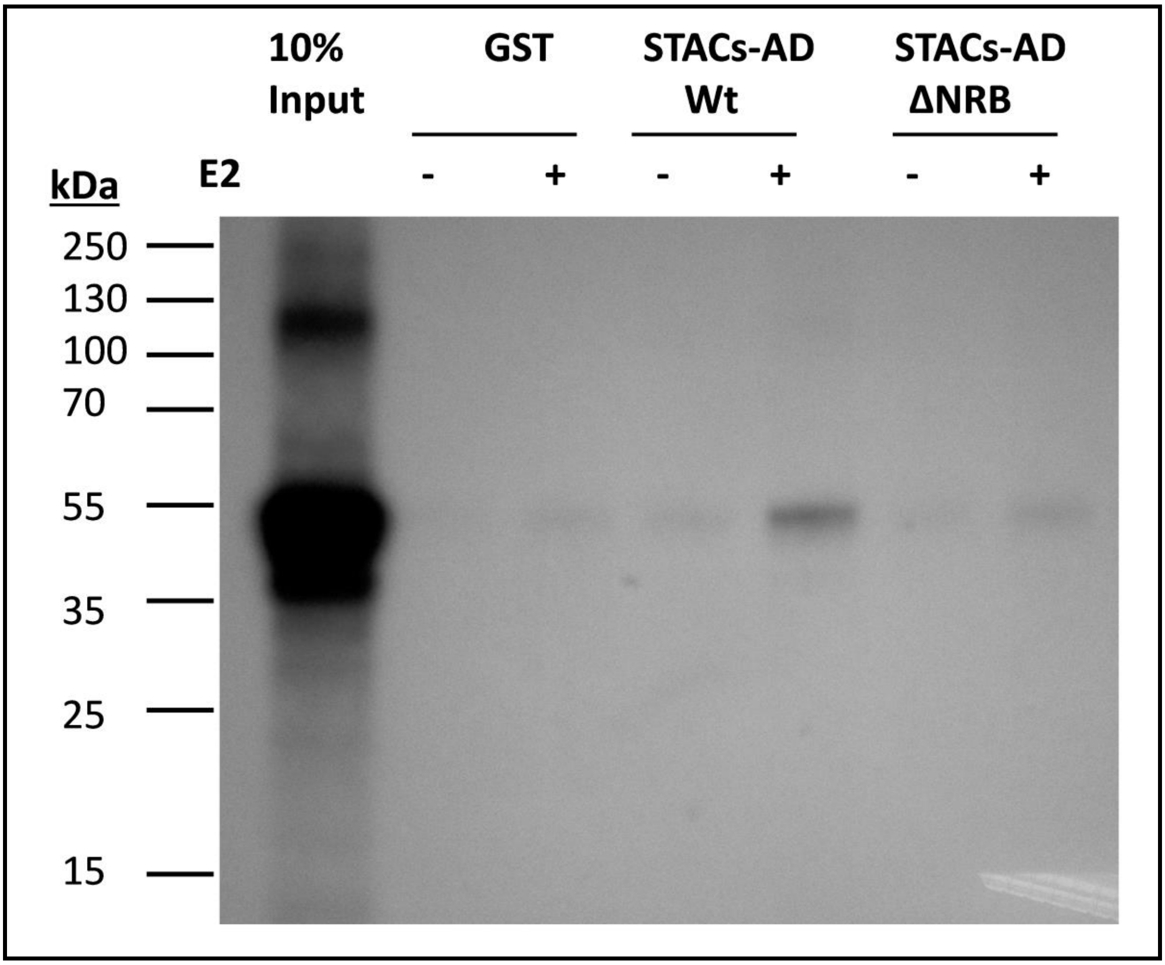

Figure S4. STACs-AD interaction with ERβ. Uncropped fluorograph of Figure **5d** (lower panel). *In vitro* expressed ERβ was bound to GST or GST-tagged STACs-AD wt or its NR-box mutant (ΔNRB); DMSO (-) or 1 µM E2 were added to determine ligand-independent or ligand-dependent interaction respectively. Complexes were co-resolved with "Input" ERβ.

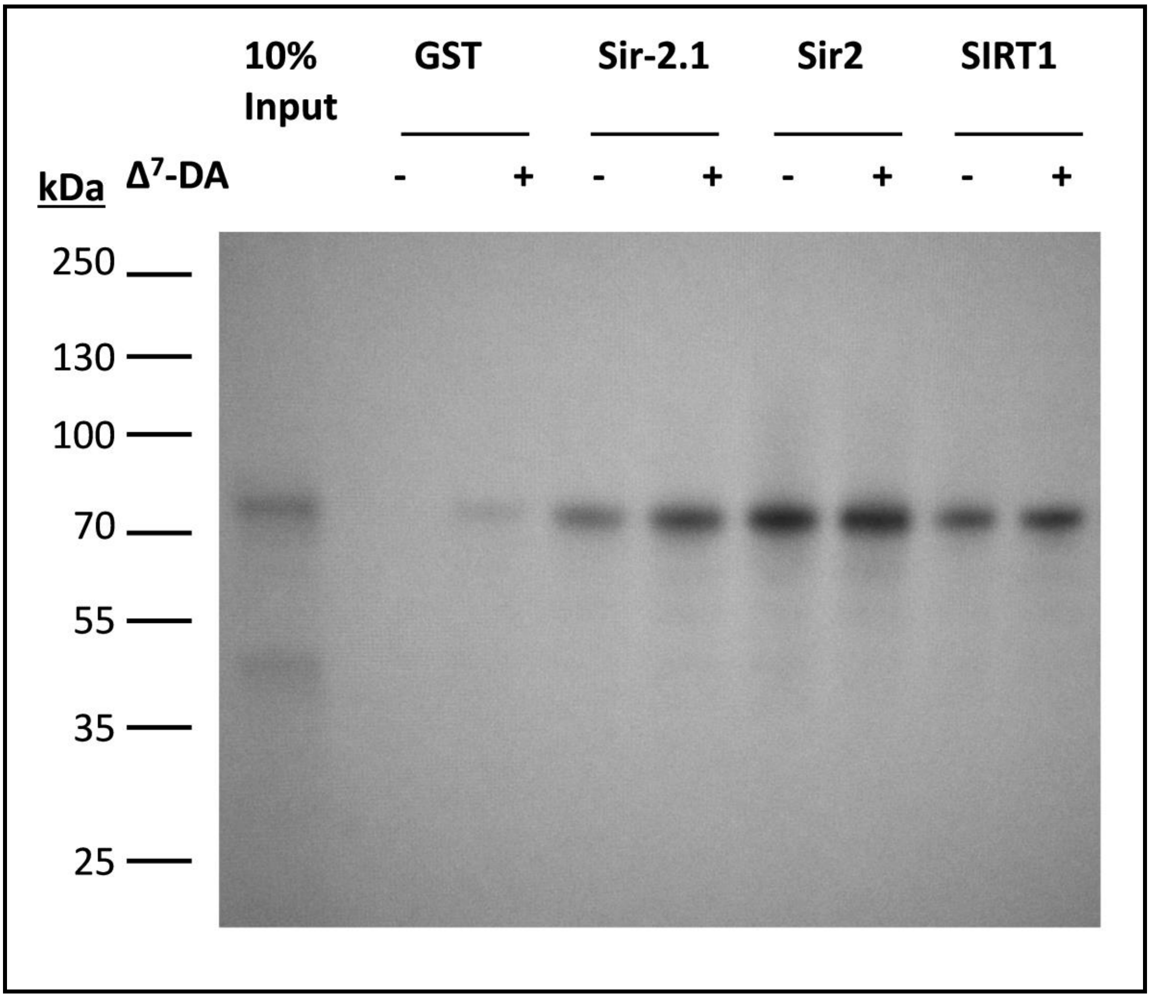

Figure S5. DAF-12 interaction with sirtuins. Fluorograph of **Figure 5e** (uncropped). *In vitro* expressed DAF-12 was bound to GST or GST-tagged Sir-2.1, Sir2 and SIRT1 (wt only) in the presence of DMSO (-) or 1 µM Δ^7-dafachronic acid (DA) to determine ligand-independent or ligand-dependent interaction respectively. Complexes were co-resolved with "Input" DAF-12.
